# Three-Dimensional Analysis Versus Two-Dimensional Slice-Based Analysis of CT for Measuring Femoral Torsion and Its Correlation to Passive Hip Range of Motion

**DOI:** 10.7759/cureus.29554

**Published:** 2022-09-25

**Authors:** Drew Ratner, Jensen G Kolaczko, Kirk Jeffers, Floor Lambers, Alexandra Orahovats, Trevor Wait, Ognjen Stevanovic, James Genuario

**Affiliations:** 1 Orthopedic Surgery, Blue Ridge Orthopedics-Easley, Easley, USA; 2 Orthopedic Surgery, Steadman Hawkins Clinic Denver, Englewood, USA; 3 Orthopedic Surgery, Orthopedic Centers of Colorado, Englewood, USA; 4 Sports Medicine, Stryker, Basel, CHE; 5 Orthopedics, Steadman Hawkins Clinic Denver, Englewood, USA

**Keywords:** femoral torsion, arthroscopy surgeon, developmental dysplasia of the hip, femoroacetabular impingement, hip preservation surgery

## Abstract

Introduction

Femoral torsion is an important anatomical consideration of the hip that has major implications on the natural motion of the hip joint. Similarly, it affects pathologic conditions of the hip, including femoroacetabular impingement, dysplasia, and/or microinstability. Femoral torsion is typically measured on two-dimensional (2D) axial CT cuts by creating the angle between the femoral neck and the posterior aspect of the ipsilateral femoral condyles. Position of the leg during imaging may affect 2D measurements. Three-dimensional (3D) analysis of a hip CT with inclusion of femoral alignment may portray the anatomy of the hip more accurately as compared to a 2D slice-based analysis of a hip CT scan. It is thought that femoral torsion measured using this system could be a more accurate and reliable means of measurement. The primary purpose of this study is to assess the differences in measuring femoral torsion with 3D modeling and analysis compared to the standard 2D slice-based approach on a CT scan. Secondarily, we attempt to determine how the passive range of motion of the hip correlates with femoral torsion measured using the 3D model versus the 2D model.

Methods

In a prospective cohort study of 20 patients, femoral torsion was assessed using both 2D analysis and 3D analysis. The differences between these measurements on each of the imaging modalities were compared. Additionally, each patient had the passive range of motion of their hip measured with a goniometer. The amount of internal and external rotation was measured with the hip in a neutral position and with the hip flexed to 90°. Acetabular version, combined version, and alpha angle were added to multivariate regression analysis to evaluate their effect versus femoral torsion alone.

Results

Femoral antetorsion measured using the standard 2D slice-based approach on CT scan was 22.1° (SD: 11.1°), which was higher (p<0.001) than that using 3D analysis (8.25°; SD: 10.5°). There was a strong correlation between femoral torsion measurements using 3D analysis and 2D analysis (R=0.91). Based on 3D analysis, there was a moderate correlation between femoral torsion and passive hip external rotation measured with the hip flexed to 90° (R=0.65, p<0.002) and with the hip in a neutral position (R=0.58, p<0.007).

Conclusion

There was a significant difference between femoral torsion measurements using the 3D analysis, which showed approximately 14° of less antetorsion on average. Additionally, rotation of the hip and femoral torsion was correlated to higher levels of antetorsion associated with more internal rotation of the hip.

## Introduction

Femoral torsion is defined as the angle between the femoral neck axis and the posterior condylar axis of the knee in the axial plane [1]. Femoral torsion is a relevant measurement that affects hip range of motion and pathology and is an important factor to consider in femoroacetabular impingement. The bony alignment of the femoroacetabular joint is one of the main static constraints to hip stability and motion. Femoral retrotorsion can exacerbate anterior impingement by decreasing the amount of internal rotation of the hip before the femoral neck comes in contact with anterior intra- and extra-articular structures. Conversely, femoral antetorsion can exacerbate posterior impingement by decreasing the amount of external rotation before the femoral neck comes into contact intra- and extra-articular structures [2]. This relationship and stress on the anterior structures is especially magnified when there is anterior undercoverage with hip instability or dysplasia [3,4]. Thus, accurate measurement of femoral torsion is important for diagnosing different hip conditions and can help guide treatment [5,6].

Currently, many believe the standard two-dimensional (2D) slice-based analysis of CT scan to be the best tool to measure femoral torsion, while some prefer MRI. There have been no studies to prove either imaging modality as the gold standard, as both CT and MRI have flaws when trying to measure femoral torsion [7,8]. When using axial 2D imaging to determine femoral torsion, the position of the leg in both the coronal plane (ab/adduction) and sagittal plane (flexion and extension) is not accounted for. Slight variations in these positions can affect the appearance of the femoral neck in single 2D axial cuts. These variations can influence femoral torsion by up to 12.5° [9]. Because of the complexity and variability of the three-dimensional (3D) anatomy of the femur, it is thought that 3D analysis of the CT scan with assessment of the neck, condylar axis, and alignment of the femur could more accurately measure femoral torsion [10].

Femoral torsion’s effect on the internal and external rotation of the hip has been extensively studied. Several studies have shown that, in general, internal rotation (both at a neutral hip position and at 90˚ of hip flexion) is greatest in hips with combined femoral antetorsion and acetabular anteversion, whereas external rotation is correspondingly least in such hips. Conversely, internal rotation was least in hips with femoral retrotorsion and acetabular retroversion, with the opposite trends observed for external rotation [7,11-15].

The primary purpose of this study is to assess the differences in measuring femoral torsion with 3D modeling and analysis compared to the standard 2D approach on a CT scan. A secondary purpose is to determine how clinical assessment of passive range of motion of the hip correlates with femoral torsion, acetabular version, combined version, and alpha angle on both 2D and 3D imaging.

## Materials and methods

We prospectively analyzed 20 patients aged 18 to 65 years with a chief complaint of hip pain. All included patients had a documented physical examination as well as 2D CT and 3D analysis (HipMap FAI analysis, Stryker, Kalamazoo) of the hip. Patients were excluded if they had previous surgery on the hip. There were no dropouts. IRB approval was obtained from the Colorado Multiple Institutional Review Board.

**Figure 1:**
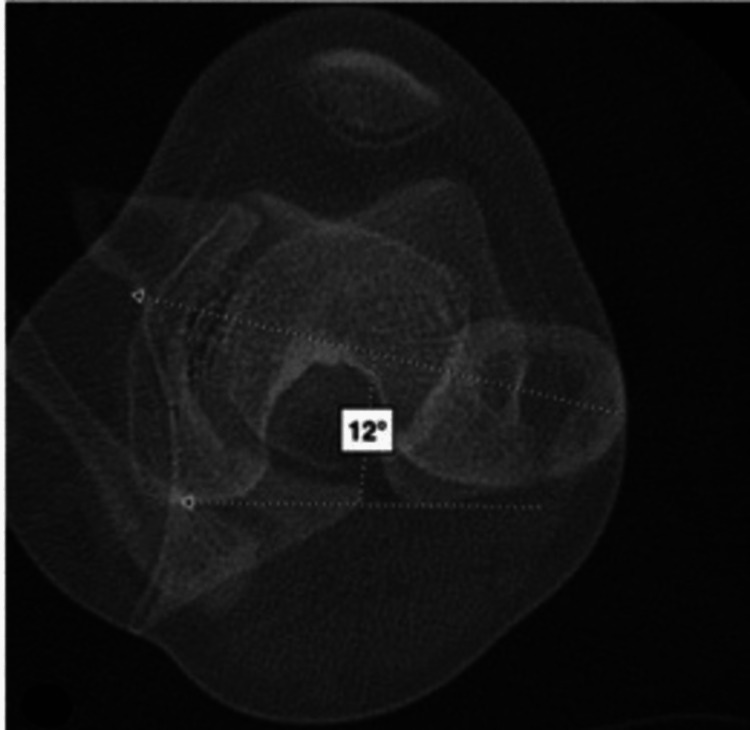
Method for measuring femoral torsion using the standard 2D slice-based approach [12]

All measurements on the 2D CT scans were performed by musculoskeletal radiologists at our institution. The reference line for the proximal femur was obtained by the technique described by Tomczak et al., in which the femoral head center was connected to the center of the greater trochanter at the base of the femoral neck [6]. This line was then superimposed over the distal femur in line with the epicondylar axis, and the angle formed with the posterior condylar line was taken as the femoral torsion (Figure 1). Femoral torsion on 3D imaging is measured as the projected (axial) angle between the femoral neck axis and the condylar axis and incorporates both neck inclination and femoral shaft rotation. The femoral neck axis is defined as the line between the center of the femoral head and the weighted center at the smallest cross-section of the femoral neck as determined in a 3D process. To correct for variation in patient position during image acquisition, the femur is aligned to the long axis as defined by the center of the femoral head and the center of the posterior condyles [8,16]. Acetabular version was measured on 2D imaging as described by Anda et al. as the angle between a line connecting the anterior acetabular margin with the posterior acetabular margin and a transverse reference line through the femoral head centers [17]. Acetabular version was measured on 3D imaging as the angle between the sagittal plane (perpendicular to the frontal plane) and a line that connects the posterior and anterior aspects of the acetabular rim on a transverse plane. The alpha angle is measured as the angle between the femoral neck axis and a line from the center of the femoral head to the point at which the bone cortex diverges from the best-fitted sphere by 1 mm. The alpha angle at 3 o’clock was used for this study.

All examinations were performed at the UC Health Steadman Hawkins Clinic Denver. If patients had the appropriate imaging, then the passive range of motion of their hip was measured in the clinic. Internal and external rotations of the hip were measured with the patient in the supine position with the hip flexed at 90°. Internal and external rotations of the hip were then measured again with the patient in the prone position and the hip in the neutral position. A digital goniometer (GemRed 82305 Digital Protractor Angle Finder Stainless Steel Ruler, GemRed, Guilin, China) was used to take the measurements, which were agreed upon by two surgeons simultaneously.

Comparisons were to be drawn on differences in accuracy of hip measurements between two types of physical examinations (prone and supine), the femoral torsion, acetabular version, combined version, and alpha angle as measured using 3D and 2D analyses. Descriptive statistics were run to describe the population by age, sex, and the side of the hip examined. Difference scores were created for the prone and supine physical examinations. Pearson product-moment correlation was run between each physical measurement type using their respective difference scores and the imaging measurements obtained using 2D and 3D analyses. A paired-samples t-test was run to compare the difference in means between the 2D and 3D torsion methods. A linear regression model was used to assess the influence of femoral torsion, acetabular version, combined version, and alpha angle. Underlying statistical assumptions were tested prior to each inferential procedure. All analyses were completed with R (R: A Language and Environment for Statistical Computing, R Foundation for Statistical Computing, Vienna, Austria). A post-hoc power analysis was performed; with a sample size of 20, alpha of 0.05, and one-tailed t-tests, the observed post-hoc power was determined to be 69.5%.

## Results

**Figure 2:**
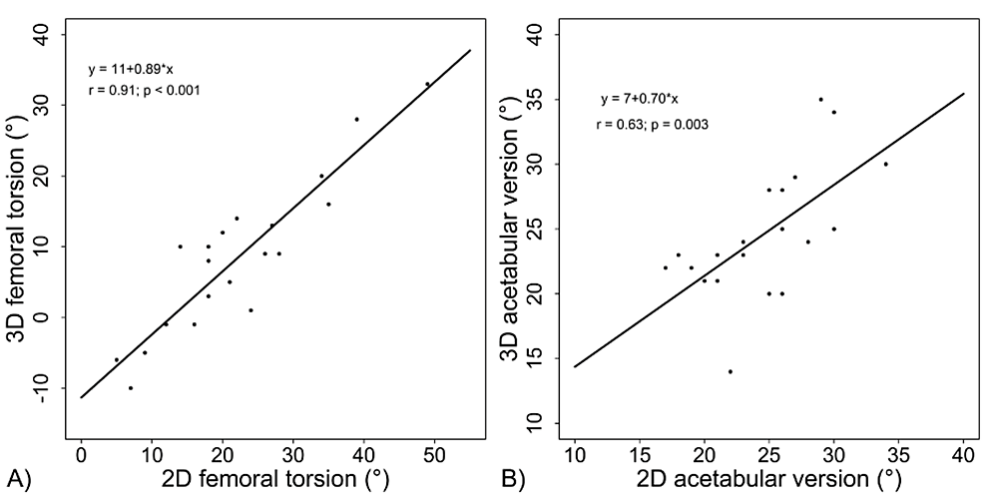
Correlation between 2D axial measurement and 3D analysis for (A) femoral torsion and (B) acetabular version 2D, two dimensional; 3D, three dimensional

A total of 20 participants were analyzed, of which 18 (90%) were female and 2 (10%) were male. The mean age of participants was 32.2 (SD=8.77). A total of 11 (55%) right hips and 9 (45%) left hips were studied. Table 1 displays the patient demographics and Table 2 displays the means and standard deviations for the hip rotation measurements for the 20 participants. Overall, 3D CT analysis had a lower mean antetorsion of 8.25° (SD=10.54) as compared to 2D CT analysis, which had a mean antetorsion of 22.10° (SD=11.12). Together, 3D and 2D CT analyses had a strong positive correlation with each other (R=0.905, Figure 2).

**Table 1:** Patient demographics

	Descriptive statistics	
Hips studied	Total	20
Age	Mean	32.2 (±8.77)
Sex	Total female (proportion)	18 (90%)
	Total male (proportion)	2 (10%)
Side studied	Total right (proportion)	11 right (55%)
	Total left (proportion)	9 left (45%)

**Table 2:** Supine and prone, internal and external rotation averages, and femoral torsion averages as measured by image type SD, standard deviation; 2D, two dimensional; 3D, three dimensional

		Mean (°)	SD (°)
Rotation			
Supine	Internal	24.55	9.85
	External	38.60	13.32
Prone	Internal	37.45	14.79
	External	37.25	12.78
Femoral torsion			
3D analysis		8.25	10.5
2D CT slice-based method		22.1	11.1

**Figure 3:**
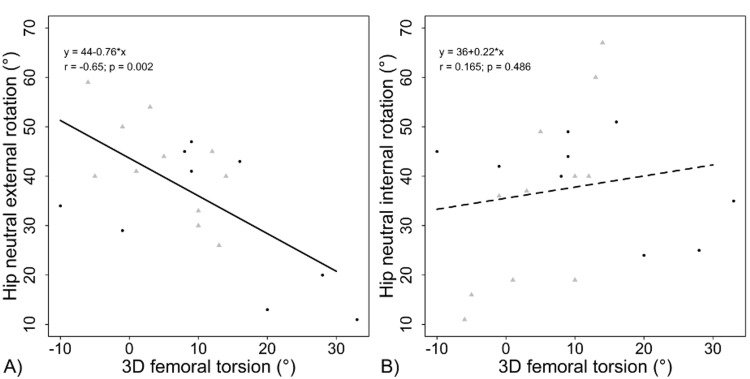
Femoral torsion is (A) correlated to external rotation and (B) not to internal rotation Black dots indicate left-sided hips and grey triangles right-sided hips 2D, two dimensional; 3D, three dimensional

The internal and external rotation of the hips were correlated with femoral torsion, acetabular version, combined version, and alpha angle measured using the 3D and 2D CT analysis. Table 3 reflects these correlations, which demonstrate that there is a significant relationship between the external rotation of the hip (measured with the hip flexed and in the neutral position) and both the 3D and 2D analyses of the torsion. This correlation is negative, demonstrating an inverse relationship. Figure 3 demonstrates the specific relationship between 3D torsion and rotation of the hip in neutral. Internal rotation did not have a significant correlation with femoral torsion. It did, however, have a statistically significant positive correlation with acetabular anteversion measured on 2D imaging. The 3D combined version reflected similar findings seen by femoral torsion alone. Alpha angle had no measurable effect on rotation.

**Table 3:** Pearson correlation coefficients between range of motion measurements (internal and external rotation while flexed and in the neutral positions) and morphological measurements (femoral torsion, acetabular version, combined version, and alpha angle) *Denotes significance at p<0.05 2D, two dimensional; 3D, three dimensional

		3D femoral torsion		2D femoral torsion		3D acetabular version		2D acetabular version		3D combined version		2D combined version		3D Alpha angle		2D Alpha angle	
	Rotation	r	p-value	r	p-value	r	p-value	r	p-value	r	p-value	r	p-value	r	p-value	r	p-value
Flexed	Internal	0.28	0.233	0.21	0.385	0.30	0.202	0.22	0.346	0.33	0.150	0.26	0.262	0.37	0.106	0.29	0.211
	External	-0.58*	0.007*	-0.51*	0.022*	-0.26	0.264	0.06	0.799	-0.56*	0.010*	-0.43	0.057	0.02	0.929	-0.08	0.738
Neutral	Internal	0.17	0.486	0.19	0.426	0.31	0.178	0.49*	0.03*	0.25	0.291	0.34	0.139	0.25	0.284	0.23	0.324
	External	-0.65*	0.002*	-0.59*	0.006*	-0.05	0.825	0.26	0.265	-0.54*	0.014*	-0.44	0.055	0.09	0.691	0.08	0.723

**Figure 4:**
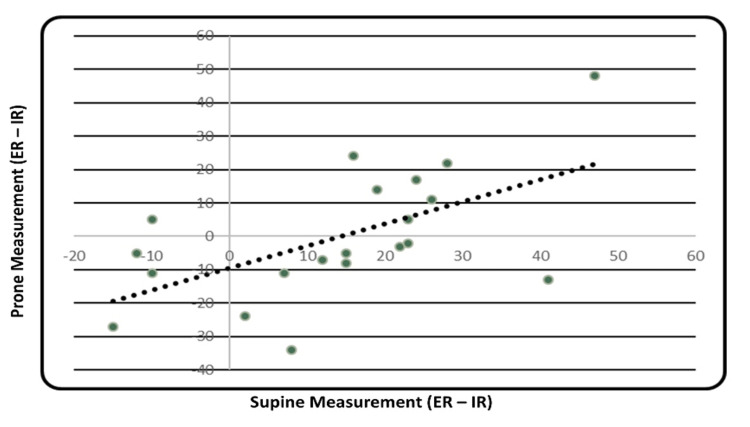
Correlation between supine and prone measurements of hip rotation

The correlation between measurement of hip rotation in neutral alignment (prone position) versus a 90° flexed alignment (supine position) was compared and shown to have a moderate correlation (R=0.58, p<0.007; Figure 4).

**Figure 5:**
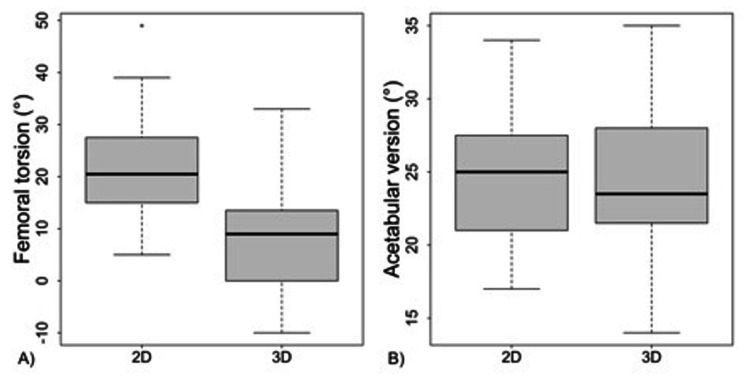
Boxplot of (A) femoral torsion and (B) acetabular version as measured on 2D axial slices or analyzed after 3D alignment 2D, two dimensional; 3D, three dimensional

**Figure 6:**
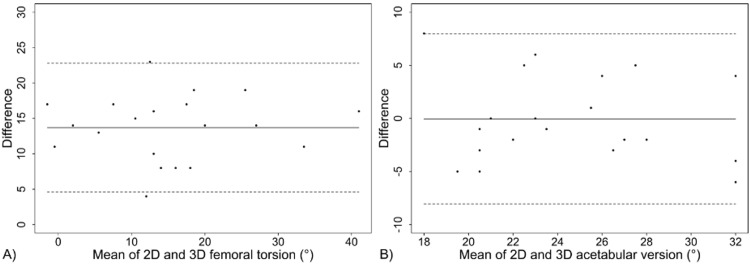
Blant-Altman plots of 2D vs 3D differences in (A) femoral torsion and (B) acetabular version 2D, two dimensional; 3D, three dimensional

Figure 5 is a box plot representation of the range of values seen in 2D versus 3D measurements of both femoral torsion and acetabular version. Figure 6 is a Blant-Altman plot of the difference in femoral torsion and acetabular version measurements on each type of imaging. From these plots, it is evident that torsion in 2D analysis differs significantly from torsion in 3D analysis by 13.85°, while there is no significant difference between acetabular version as measured by 2D versus 3D imaging (average difference 0.05°).

## Discussion

The primary finding in this study is that there was a significant difference in the measurement of femoral torsion between the currently used 2D slice-based analysis of CT scan and the proposed 3D analysis. On average, the 3D analysis shows 13.85° less antetorsion than the 2D approach (p<0.001), but the approaches were highly correlated (R=0.905). This suggests the measurements are similar but offset in range. We believe that this difference is seen for three primary reasons. First, when measuring femoral torsion using the current standard CT methods, it is important to indicate how the radiologist is measuring the angle of the proximal femur. Schmaranzer et al. have shown that the measurement for femoral torsion can differ greatly on the basis of technique used for this proximal reference line [18]. In our study, the radiologists used the technique described by Tomczak et al., in which the femoral head center was connected to the center of the greater trochanter at the base of the femoral neck. This line was then superimposed over the distal femur in line with the epicondylar axis, and the angle formed with the posterior condylar line was taken as the femoral torsion [6]. By comparison, with the 3D analysis, engineers use the “fitting process” described in the Methods section to analyze the angle of the proximal femur, which we believe more accurately depicts the true torsion of the femur.

A second reason that we believe the 3D analysis shows lower values for torsion is due to the way in which 3D analysis aligns the proximal and distal femur to account for the true anatomy. The current standard method of measuring femoral torsion is to overlay the femoral head and neck over the condyles of the knee with the center of the femoral head along the epicondylar axis of the knee and obtain femoral torsion in that orientation. In the 3D analysis we used for this study, femoral torsion is measured by aligning the center of the femoral head with the posterior condyles of the distal femur. This technique resulted in less antetorsion measured (Figure 4).

The third reason for the discrepancy in measurements between standard 2D CT and 3D analysis is that 3D analysis accounts for the leg positioning inside the CT scanner. It has been shown in the Stryker Orthopaedics Modeling and Analytics System (SOMA) that more flexion and abduction of the hip can lead to increasing measurements of torsion (Figure 5). Note the difference in 2D rotational profile of the proximal femur with these changes. The 3D analysis that we employed attempts to compensate for leg positioning.

An additional finding in this study is that there is a significant correlation between external rotation of the hip on physical examination and torsion of the femur, whether measured with the hip at 90° of flexion or in a neutral position. Several previous studies have also indicated a significant association between internal rotation of the hip and femoral torsion. Chadayammuri et al. looked specifically at antetorsion of the femur and acetabular anteversion and showed that as these variables increased, internal rotation increased significantly [12]. Audenaert et al. showed that 75% of the observed variance among measurements of internal rotation of the hip would be attributed to femoral head asphericity, acetabular coverage, and femoral torsion [11]. Alpha angle (asphericity) also demonstrated no significant difference in motion in this study. It is unclear to us why external rotation had a more significant correlation with torsion compared to internal rotation and alpha angle in our study.

There are several limitations to our study. First, because we only order 3D analysis for our surgical patients, all the patients in our study ended up undergoing an arthroscopic procedure of their hip. For that reason, they all had some type of painful pathology (femoroacetabular impingement or microinstability) that was being addressed arthroscopically. This could affect range of motion, which taken preoperatively in all instances may have affected outcomes as shown in the study by Tokpinar et al. [19]. Kelly et al. showed that increased antetorsion led to increased internal rotation of the hip, but, additionally, arthroscopic decompression results in improvement of internal rotation at 90° of hip flexion in impingement-related disease of the hip [14]. Analyzing a control group of asymptomatic patients for comparison could have helped in making the range of motion conclusions more generalizable. Second, we had a relatively small sample size of 20 patients, of whom 18 were female. A larger study group with a more evenly distributed male-to-female ratio could also help make the conclusion more generalizable. Finally, all our physical examination measurements were done on awake patients, and therefore pain and/or muscular contraction could influence hip rotation.

## Conclusions

In conclusion, 3D analysis of the CT scans showed a good correlation with standard analysis of CT scan and physical examination of the hip, but overall revealed decreased antetorsion of approximately 14° in our study population. This is a considerable difference that could have important implications when making operative decisions on both hip impingement and instability. This study illustrates a significant difference between femoral torsion measurements using the standard 2D CT slice-based approach and 3D analysis. However, further investigative studies are needed to more elucidate which method more closely depicts true torsion.
